# Pregnancy-related complications in patients with endometriosis in different stages

**DOI:** 10.1186/s40834-024-00280-0

**Published:** 2024-05-14

**Authors:** Khadijeh Shadjoo, Atefeh Gorgin, Narges Maleki, Arash Mohazzab, Maryam Armand, Atiyeh Hadavandkhani, Zahra Sehat, Aynaz Foroughi Eghbal

**Affiliations:** 1 Reproductive Biotechnology Research Center, Avicenna Research Institute, ACECR, Tehran, Iran; 2 Infertility Clinic, Avicenna Research Institute, ACECR, Tehran, Iran; 3 Department of Epidemiology, School of Public Health, Iran University of Medical Sciences, Tehran, Iran; 4 Urmia University of Medical Sciences, Urmia, Iran

**Keywords:** Endometriosis, Fertility, Neonatal, Pregnancy complication, Outcome

## Abstract

**Background:**

Endometriosis is one of the most common and costly diseases among women. This study was carried out to investigate pregnancy outcomes in women with endometriosis because of the high prevalence of endometriosis in reproductive ages and its effect on pregnancy-related complications outcomes.

**Methods:**

This was a cross-sectional study performed on 379 pregnant women with endometriosis who were referred to the endometriosis clinic of the Avicenna Infertility Treatment Center from 2014 to 2020. Maternal and neonatal outcomes were assessed for the endometriosis group and healthy mothers. The group with endometriosis was further divided into two groups: those who underwent surgery and those who either received medication alone or were left untreated before becoming pregnant. The analysis of the data was done using SPSS 18.

**Results:**

The mean age of the patients was 33.65 ± 7.9 years. The frequency of endometriosis stage (*P* = 0.622) and surgery (*P* = 0.400) in different age groups were not statistically significant. The highest rates of RIF and infertility were in stages 3 (*N* = 46, 17.2%) (*P* = 0.067), and 4 (*N* = 129, 48.3%) (*P* = 0.073), respectively, but these differences were not statistically different, and the highest rate of pregnancy with ART/spontaneous pregnancy was observed in stage 4 without significant differences (*P* = 0.259). Besides, the frequency of clinical/ectopic pregnancy and cesarean section was not statistically different across stages (*P* > 0.05). There is no significant relationship between endometriosis surgery and infertility (*P* = 0.089) and RIF (*P* = 0.232). Most of the people who had endometriosis surgery with assisted reproductive methods got pregnant, and this relationship was statistically significant (*P* = 0.002) in which 77.1% (*N* = 138) of ART and 63% (*N* = 264) of spontaneous pregnancies were reported in patients with endometriosis surgery. The rate of live births (59.4%) was not statistically significant for different endometriosis stages (*P* = 0.638). There was no stillbirth or neonatal death in this study. All cases with preeclampsia (*N* = 5) were reported in stage 4. 66.7% (*N* = 8) of the preterm labor was in stage 4 and 33.3% (*N* = 4) was in stage 3 (*P* = 0.005). Antepartum bleeding, antepartum hospital admission, preterm labor, gestational diabetes, gestational hypertension, abortion, placental complications and NICU admission were higher in stage 4, but this difference had no statistical difference.

**Conclusion:**

Endometriosis is significantly correlated with infertility. The highest rates of RIF and infertility are observed in stages 3 and 4 of endometriosis. The rate of pregnancy with ART/spontaneous pregnancy, preterm labor, preeclampsia and pregnancy-related complications is higher in stage 4. Most of the people who had endometriosis surgery with assisted reproductive methods got significantly pregnant. Clinical/ectopic pregnancy, cesarean sections, and live birth were not affected by the endometriosis stages.

## Introduction

The presence of endometrial-like glandular tissue, stroma, or endometrial tissue outside the uterine cavity is known as endometriosis, a chronic gynecological disease that affects 30 to 50% of infertile women [1]. Endometriosis commonly affects various parts of the female reproductive system, including the pelvic area, ovaries, posterior cul-de-sac, uterine ligaments, pelvic peritoneum, rectovaginal septum, cervix, vulva, vagina, as well as the intestines and urinary system. Endometriosis can cause symptoms like infertility, dysmenorrhea, and chronic pelvic inflammatory disease, which can worsen pain, dyspareunia, and painful bowel movements, ultimately lowering the quality of life for the affected woman [2, 3–12, 13]. Laparoscopic surgery is both the standard surgical procedure and the best treatment for endometriosis [14]. However, endometriosis remains a problematic issue due to its negative impact on ovarian reserve and the recurrence rate of 40–50% after 5 years of surgery [15, 16]. Numerous studies have shown the negative effects of endometriosis on pregnancy, including the increase in preterm labor, placental abruption and cesarean delivery, preeclampsia, placental problems and postpartum hemorrhage, premature rupture of membranes (PROM), preterm birth, small for gestational age (SGA), NICU admission, neonatal mortality and morbidity, and hypertensive disorders of pregnancy (HDP) with low birth weight (LBW) [2, 5, 6, 17]. Since the effects of endometriosis on the course of pregnancy are still controversial, this work aimed to first identify the negative effects of endometriosis on pregnancy and then determine whether laparoscopic surgery or other drug interventions before pregnancy were beneficial.

## Materials and methods

This cross-sectional study was carried out on 379 pregnant women with a history of endometriosis and pregnancy who were referred to the endometriosis clinic of the Avicenna Infertility Treatment Center between January 2014 and January 2020. This study was approved by the Ethics Committee of Avicenna Infertility Treatment Center (IR.ACECR.AVICENNA.REC.1398.031) in accordance with the tents of the Declaration of Helsinki, and the patient’s oral and written consent was obtained to ensure that they participated in the study voluntarily. Specific means of identifying endometriosis were approved after laparoscopic surgery with pathologic confirmation, magnetic resonance imaging (MRI), ultrasound imaging, and clinically confirmed presence of symptoms. Exclusion criteria were less than 22 weeks of gestation at the time of delivery, fetal malformations, and incomplete medical files. Maternal and neonatal outcomes were assessed for the endometriosis group and healthy mothers. The group with endometriosis was further divided into two groups: those who underwent surgery and those who either received medication alone or were left untreated before becoming pregnant. A history of laparoscopic surgery or other surgeries and hormonal therapies (oral contraceptive pills, progestin, and gonadotropin-releasing hormone agonists) were obtained from the patient’s medical files. Maternal characteristics in this study included maternal age, parity, pre-pregnancy weight and BMI, pre-pregnancy blood pressure, chronic hypertension, diabetes mellitus (DM), cholestasis, and assisted reproductive technology (ART). Outcomes evaluated included gestational age, ectopic pregnancy, clinical pregnancy, mode of delivery, antepartum hemorrhage, antepartum hospitalization, preterm labor (< 37 weeks of gestation), labor dystocia, gestational diabetes mellitus (GDM), gestational hypertension, gestational cholestasis, placental abruption and placenta previa, PROM, and abortion. Neonatal characteristics included birth weight, height, SGA, stillbirth, neonatal death, and NICU admission.

### Statistical analysis

The data were analyzed using SPSS 18. Normality was checked using the Kolmogorov-Smirnov test. Continuous variables with a normal distribution were summarized as mean and standard deviation and compared between the two groups using an independent t-test. Categorical variables were presented as frequency and percentage to be compared between the two groups using either the Fisher’s exact test or the chi-square (*x*^2^) test. The significance level was defined as *p* < 0.05.

## Results

During the study period, all 379 women with a mean age of 33.65 ± 7.9 years underwent treatment and were followed until a negative pregnancy test or the end of the pregnancy. The mean marriage duration was 9.72 ± 4.71 years. In this study, 16.1% of the people were in the age group of 25–30 years, 35.6% were in the age group of 30–35 years, and the rest (92.3%) belonged to the age group of more than 40 years. The age group with the highest number of surgeries for endometriosis is 35–40 years. The age group of 25–30 years experiences the highest incidence of stage 1 endometriosis, while the age group of 30–35 years has the highest occurrence of stage 2. Additionally, the age group of 30–35 years also has the highest number of individuals with stage 3, while the age group of 35–40 years has the highest number of people with stage 4 (Table 1). The majority of patients in stage 4 needed surgery (89.9%) (Table 2).

**Table 1:** Frequency of endometriosis stage and surgery in different age groups

Age group	Endometriosis stage				Endometriosis surgery		Total
	1	2	3	4	Yes	No	
25–30 yrs	2) 50.0%)	2(16.7%)	15(15.6%)	42(15.7%)	41(15.5%)	20(17.4%)	61(16.1%)
30–35 yrs	1(25.0%)	5(41.7%)	41(42.7%)	88(33.0%)	88(33.3%)	47(40.9%)	135(35.6%)
35–40 yrs	1(25.0%)	4(33.0%)	34(35.4%)	115(43.1%)	114(43.2%)	40(34.8%)	154(40.6%)
> 40 yrs	0(0.0%)	1(8.3%)	6(6.3%)	22(8.2%)	21(8.0%)	8(7.0%)	29(7.7%)
Total	4(100%)	12(100%)	96(100%)	267(100%)	264(100%)	115(100%)	379(100%)
P-value = 0.622					P-value = 0.400		

**Table 2:** Frequency of surgery in different endometriosis stages

Endometriosis stage	Endometriosis surgery		Total
	Yes	No	
1	0	4(100.0%)	4(100.0%)
2	0	12(100.0%)	12(100.0%)
3	24(25.0%)	72(75.0%)	96(100.0%)
4	240(89.9%)	27(10.1%)	267(100.0%)
Total	264(69.7%)	115(30.3%	379(100.0%)
P-value	< 0.001		

According to the information in Table 3, the highest rate of RIF and infertility was in stage 3 (*N* = 46, 17.2%) (*P* = 0.067), and 4 (*N* = 129, 48.3%) (*P* = 0.073), respectively but these differences were not statistically significant. Also, the highest rate of pregnancy with ART/spontaneous pregnancy was observed in stage 4 without significant differences (*P* = 0.259). Besides, the frequency of clinical/ectopic pregnancy and cesarean sections was not statistically different across stages (*P* > 0.05) (Table 4).

**Table 3:** Frequency of infertility, RIF, pregnancies, and cesarean sections in different endometriosis stages

Stage	Infertility, %		RIF, %		Pregnancy, %		Clinical pregnancy, %		Ectopic pregnancy, %		Cesarean section, %		Total, %
					ART	Spontaneous							
	Yes	No	Yes	No			Yes	No	Yes	No	Yes	No	
1	0	4 (100%)	0	4 (100)	1 (25)	3 (75)	4 (100)	0	0	4 (100.0)	2 (50)	2 (50)	4 (100)
2	6 (50.0)	6 (50.0%)	4 (33.3)	8 (66.7)	6 (50)	6 (50)	12 (100)	0	0	12 (100.0)	8 (66.7)	4 (33.3)	12 (100)
3	38 (39.6)	58 (60.4%)	9(9.4)	87 (90.6)	38 (39.6)	58 (60.4)	94 (97.9)	2 (2.1)	2 (2.1)	94 (97.9)	60 (62.5)	36 (37.5)	96 (100)
4	129 (48.3)	138 (51.7%)	46 (17.2)	221 (82.8)	134 (50.2)	133 (49.8)	265 (99.3)	2 (0.7)	2 (0.7)	265 (99.3)	187 (70.0)	80 (30.0)	267 (100)
Total	173 (45.6)	206 (54.4%)	59 (15.6)	320 (84.4)	180 (25)	203 (75)	375 (98.9)	4 (1.1)	4 (1.1)	375 (98.9)	257 (67.8)	122 (32.2)	379 (100)
P-value	0.067		0.073		0.259		0.710		0.379		0.487		0.487

**Table 4:** Frequency of infertility, RIF, and pregnancy in people with a history of endometriosis surgery

Variable		Infertility		RIF	Pregnancy		Total
		Yes	No		ART	Spontaneous	
Endometriosis surgery	Yes	127(73.4%)	137(66.5%)	44(74.6%)	138(77.1%)	126(63%)	264(69.7%)
	No	46(26.6%)	69(33.5%)	15(25.4%)	41(22.9%)	74(37%)	115(30.3%)
Total		173(100%)	206(100%)	59(100%)	179(100%)	200(100%)	379(100%)
P-value		0.089		0.232	0.002		

There is no significant relationship between endometriosis surgery and infertility (*P* = 0.089) and RIF (*P* = 0.232). Most of the people who had endometriosis surgery with assisted reproductive methods got pregnant, and this relationship was statistically significant (*P* = 0.002) in which 77.1% (*N* = 138) of ART and 63% (*N* = 264) of spontaneous pregnancies were reported in patients with endometriosis surgery (Table 3).

The rate of live births (59.4%) was not statistically significant by different endometriosis stages (*P* = 0.638) (Table 5).

**Table 5:** Frequency of live births in different endometriosis stages

Stage	Live birth rate		Total
	Yes	No	
1	2(50%)	2(50%)	4(100%)
2	7(58.3%)	5(41.7%	12(100%)
3	52(54.2%)	44(45.8%)	96(100%)
4	164(61.4%)	103(38.6%)	267(100%)
Total	225(59.4%)	154(40.6%)	379(100%)
P-value	0.638		

There was no stillbirth or neonatal death in this study. All cases with preeclampsia (*N* = 5) were reported in stage 4. Additionally, 66.7% (*N* = 8) of the preterm labor were in stage 4 and 33.3% (*N* = 4) were in stage 3 in which this difference was statistically significant (*P* = 0.005). Antepartum bleeding (70%), antepartum hospital admission (75.9%), preterm labor (66.7%), gestational diabetes (80%), gestational hypertension (85.7%), abortion (71.4%), placental complications (66.7%) and NICU admission (71%) were higher in stage 4 but this difference had no statistical difference (Table 6).

**Table 6:** Comparison of pregnancy-related outcomes in different endometriosis stages

Variable		Endometriosis stage, N (%)				P-value
		1	2	3	4	
Antepartum bleeding	Yes	1 (2.5)	1 (2.5)	10 (25)	28 (70)	0.065
	No	7 (3.8)	6 (3.2)	18 (9.7)	154 (83.2)	
Antepartum hospital admission	Yes	1 (3.4)	1 (3.4)	5 (17.2)	22 (75.9)	0.094
	No	7 (3.6)	6 (3.1)	22 (11.3)	160 (82.1)	
Preterm labor	Yes	-	-	4 (33.3)	8 (66.7)	0.005
	No	8 (3.8)	7 (3.3)	24 (11.3)	174 (81.7)	
Labor dystocia	Yes	-	-	1 (50)	1 (50)	0.197
	No	8 (3.6)	7 (3.2)	26 (11.7)	181 (81.5)	
Gestational diabetes	Yes	-	-	5 [20]	20 (80)	0.109
	No	8 [4]	7 (3.5)	22 (11.1)	162 (81.4)	
Gestational hypertension	Yes	1 (14.3)	-	-	6 (85.7)	0.156
	No	7 (3.2)	7 (3.2)	27 (12.4)	176 (81.1)	
Gestational cholestasis	Yes	-	-	2 (50)	2 (50)	0.117
	No	8 (3.7)	6 (2.7)	25 (11.4)	180 (82.2)	
Abortion	Yes	-	1 (4.8)	5 (23.8)	15 (71.4)	0.114
	No	8 (3.9)	6 [3]	22 (10.8)	167 (82.3)	
Placenta previa	Yes	-	-	1 (50)	1 (50)	0.197
	No	8 (3.6)	7 (3.2)	26 (11.7)	181 (81.5)	
Preeclampsia	Yes	-	-	-	5 (100)	0.350
	No	8 (3.7)	7 (3.2)	27 (12.3)	177 (80.8)	
Placental complication	Yes	-	-	3 (33.3)	6 (66.7)	0.169
	No	8 (3.7)	7 (3.3)	24 (11.2)	176 (81.9)	
NICU admission	Yes	1 (3.2)	1 (3.2)	7 (22.6)	22 (71)	0.067
	No	7 (3.7)	6 (3.2)	18 (9.5)	159 (83.7)	

## Discussion

Women with endometriosis have lower fertility rates than ever before, but many of them are still able to give birth because of advancements in IVF and intracytoplasmic sperm injection (ICSI) technology. This cross-sectional research was conducted to examine maternal and neonatal outcomes in endometriosis patients with a history of pregnancy referred to the Avicenna Infertility Treatment Center between January 2014 and January 2020. Patients with endometriosis had a live birth rate of 54.9% Endometriosis is a common cause of infertility, and ART can help patients become pregnant. Despite these interventions, some studies have shown poor pregnancy outcomes in patients with endometriosis. Poor oocyte and embryo quality and impaired endometrial receptivity have been suggested as potential causes of poor clinical outcomes. Burghaus et al. Endometriosis risk factors have been identified as age at menarche, length of each menstrual cycle, length of menstrual years, number of pregnancies, miscarriages, and smoking [7].

Hardiman et al. concluded that premenstrual spotting lasting more than two days is significantly associated with endometriosis, with a higher predictive rate than painful menstruation and painful intercourse [8]. It may be more difficult to distinguish between the effects of endometriosis on pregnancy complications and the assisted reproductive process if many endometriosis-affected women use ART techniques during their pregnancies [9]. According to studies, there is no established association between endometriosis and preeclampsia, meaning that some studies report an increased risk of preeclampsia after endometriosis, while other research reports no change and other research reports a decreasing pattern [5].

Pérez-López et al. found a significant association between endometriosis and gestational diabetes mellitus [10]. Maggiore et al. found in 2016 that there is a significant connection between endometriosis and placenta previa. Furthermore, this association is not related to spontaneous insemination or laboratory-assisted reproductive techniques and occurs in both cases. In this context, fetal malformations and cesarean sections can be attributed to placenta previa [11]. There is a significant association between endometriosis, and cesarean sections and low birth weight in spontaneous fertilization, but no association has been found in ART pregnancies [6]. Also, Lim et al. found that women diagnosed with endometriosis exhibited a significantly higher incidence of unfavorable pregnancy outcomes in comparison to their counterparts who did not have endometriosis. These unfavorable outcomes associated with endometriosis encompassed preterm labor, preterm birth, preeclampsia, fetal growth restriction, placenta previa, placental abruption, stillbirth, antepartum, and postpartum bleeding. Furthermore, they also demonstrated an augmented risk of blood transfusion, uterine artery embolization, and cesarean hysterectomy in the group of women with endometriosis as opposed to the group without this condition [18]. Besides, Miura et al. disclosed that there was a heightened incidence of postpartum hemorrhage and placenta previa in the group diagnosed with endometriosis. Nonetheless, the other maternal and neonatal consequences exhibited no significant disparity among patients with/without endometriosis [19]. Borisova et al. reported that even though patients with endometriosis may achieve pregnancy after undergoing assisted reproductive technologies, they still face a significantly elevated risk of obstetric complications. These complications include, but are not limited to, miscarriage, preterm birth, preeclampsia, placental abnormalities, hemorrhage during labor, the birth of infants who are small for their gestational age, stillbirth, and a higher incidence of cesarean section. Furthermore, it is important to note that acute complications specific to endometriosis can manifest during pregnancy, and in most cases, surgical intervention becomes necessary to address this condition [20].

Based on the aforementioned studies, the findings of our study were consistent in the majority of respects, and the novelty of our investigation lies in the evaluation of various stages of endometriosis, which holds significance as a considerable number of patients seek the assistance of pertinent clinics during the final stages. Consequently, understanding the adverse effects at the stage of interest can provide clinicians with valuable insights into effectively addressing the patients’ status.

## Conclusion

Endometriosis is significantly correlated with infertility. The highest rates of RIF and infertility are observed in stages 3 and 4 of endometriosis. The rate of pregnancy with ART/spontaneous pregnancy, preterm labor, preeclampsia, and pregnancy-related complications is higher in stage 4. Most of the people who had endometriosis surgery with assisted reproductive methods got significantly pregnant. Clinical/ectopic pregnancy, cesarean sections and live birth were not affected by endometriosis stages.

## Data Availability

The data used in this study can be send after formal and reasonable request to the corresponding author.
